# Gut *Proteobacteria* glycine metabolism regulates neuroplasticity, motivation, and reinstatement of cocaine self-administration in mice

**DOI:** 10.1080/19490976.2026.2693397

**Published:** 2026-06-26

**Authors:** Susana Delgado Ocaña, Guadalupe Herrera, Daniel Guzmán, David W. Self, Santiago Cuesta

**Affiliations:** a Department of Cell Biology and Neuroscience, Rutgers, The State University of New Jersey, Piscataway, NJ, USA; b Department of Psychiatry, University of Texas Southwestern Medical Center, Dallas, TX, USA; c Rutgers Addiction Research Center, Brain Health Institute, Rutgers Health, Piscataway, NJ, USA

**Keywords:** Cocaine, *Proteobacteria*, addiction, glycine, gut–brain axis, proteomics

## Abstract

Addiction is a chronic and relapsing disorder that affects millions of people worldwide; nonetheless, currently available FDA-approved treatments are limited in number and effectiveness. In past years, the gut–brain axis has emerged as a key modulatory factor associated with different psychiatric disorders, including addiction. Working in mice, we have shown that cocaine exposure alters the composition of the gut microbiome, increasing the abundance of *Proteobacteria*. This microbial shift, in turn, leads to a depletion in host glycine levels, altering cocaine-induced transcriptional changes in the Nucleus Accumbens (NAc) and facilitating the development of behavioral sensitization and conditioned place preference. Among the behavioral models to study psychostimulant use disorders, cocaine self-administration (SA) remains the most translational. Therefore, here we investigated whether *Proteobacteria*-induced glycine depletion can affect cocaine SA in mice. Using the human *Escherichia coli* HS and the glycine-uptake-deficient mutant *E. coli* HS Δ*CycA*, we build upon our previous findings and demonstrate that the ability of gut *Proteobacteria* to use glycine during cocaine SA shapes the trajectory and long-term neurobehavioral plasticity induced by the drug. Furthermore, we show that this bacterial-induced glycine depletion impacts the NAc proteome, altering its vulnerability to undergo molecular adaptations across different stages of the SA paradigm. Altogether, our findings show that the gut microbiome, and particularly the *Proteobacteria* phylum, is a crucial factor influencing short and long-term adaptation underlying motivation and cocaine-seeking behaviors.

## Introduction

Addiction is a chronic, relapsing disorder characterized by compulsive drug seeking and use despite adverse consequences. Addiction and substance use disorders (SUDs) affect more than 27 million people in the United States, with an estimated $740 billion annual cost.^1-7^ Unfortunately, while some pharmacological and behavioral approaches to treatment are available, most have relatively low success rates,^8^ making the development of new therapeutics a high priority.^9^

In the past years, the gut microbiome has emerged as a novel modulator of many neurological disorders in humans and animal models, including addiction.^10-19^ This intestinal microbiome is the largest and most complex ecosystem of microorganisms, including archaea, viruses, fungi, and bacteria. Among these members, bacteria are the most prominent population.^20^ Environmental factors, as well as the host genetics, will give rise to a unique and individual adult microbiome.^21^ Even though this mature microbiome is mostly stable, it is not static and can be altered by changes in environmental conditions and health disorders.^22^ These short-term variations can modify normal gut physiology and metabolite production, leading to changes in host homeostasis, metabolism, and gene expression that can result in long-lasting effects.^21^ This is also true during brain pathology and psychostimulant use, where alterations in the gut microbiome composition have been shown to influence addiction-like behaviors and drug-induced plasticity,^13^ ^,^ ^15^ ^,^ ^16^ ^,^ ^18^ ^,^ ^23-28^ positioning the gut microbiome as a factor that can influence substance use disorders. Lately, many advances have been made toward understanding the molecular mechanisms mediating the communication between the gut and the brain; however, there is still an important gap in the knowledge regarding how specific members of the gut microbiome affect cocaine use disorders (CUDs).^15^

Working in animal models of addiction, we identified a specific metabolic-mediated mechanism by which the gut microbiome affects neurobiological circuits involved in CUDs.^16^ Using sensitization and conditioned place preference, we found that cocaine-induced increases in gut norepinephrine facilitate expansion of the phylum *Proteobacteria*, a microbial change that has been consistently reported across independent clinical and preclinical studies.^29-32^ We also demonstrated that, by consuming glycine as a carbon and nitrogen source, this phylum reduces its availability in the host, altering cocaine-induced plasticity.^16^ In the central nervous system, glycine acts as both an inhibitory signal in the spinal cord and brainstem, and as a co-agonist at NMDA receptors in forebrain regions modulating glutamatergic transmission and synaptic plasticity.^33^ Accordingly, we demonstrated that this glycine reduction leads to transcriptomic alterations in dopaminergic and glutamatergic synaptic pathways in the Nucleus Accumbens (NAc), a key brain region involved in motivation, reward, and addiction,^34^ potentiating cocaine-induced sensitization and conditioned place preference.^16^

Here, we decided to further characterize this molecular mechanism during cocaine self-administration (SA), the most translational behavioral model used to study addiction.^35-37^ Unlike passive drug exposure models, SA captures core features of human addiction, including voluntary drug intake, motivation to obtain the drug, escalation of use, and relapse-like behavior. Using the human glycine-uptake-deficient mutant (*Escherichia coli* HS Δ*CycA*), we build on our previous findings and demonstrate that the ability of *Proteobacteria* to use glycine during cocaine SA can shape the trajectory and long-term neurobehavioral plasticity induced by the stimulant in mice. Furthermore, we show that the bacterial-induced glycine depletion impacts the NAc proteome, altering its vulnerability to undergo molecular adaptations across different stages of the SA paradigm. Altogether, our findings show that the gut microbiome contributes to CUD and that altering glycine metabolism in *Proteobacteria* can be used to reshape both short- and long-term adaptations driving motivation and cocaine seeking.

## Materials and methods

### Mice

Adult male mice C57BL/6 (postnatal day, PND, 75 ± 15) were kept in specific pathogen-free conditions, housed in individual cages at a constant room temperature of 22 °C–25 °C, on a 12 h reverse light/dark cycle, humidity (45%–75%), with standard chow and water available *ad libitum*. All animals were obtained from Jackson Laboratory and acclimatized for 1 week before being used for experiments. All animal protocols were approved by the Institutional Animal Care and Use Committees of the University of Texas Southwestern Medical Center and Rutgers, the State University of New Jersey, and maintained in accordance with the guidelines of the National Institutes of Health.

### Food training

To ensure consistent acquisition of operant responding and to synchronize the onset of cocaine self-administration with the timing of bacterial colonization, prior to cocaine self-administration, all mice underwent a food training. For this purpose, animals were placed on a controlled feeding regimen (approximately 1.5 g of chow per day) to limit weight gain. Over six consecutive days, they participated in 2-h training sessions during which they learned to press the active lever to obtain 20 mg sucrose pellets, until they achieved the acquisition criterion of 60 successful responses. At least one day before surgical catheterization, mice were given free access to food. Following surgery, they were allowed a recovery period of one week before behavioral testing commenced.^36^

### Surgery

One week before bacterial inoculation, all animals were implanted with an indwelling catheter in the jugular vein under anesthesia with oxygen/isofluorane (1.5%–2.0%) ventilation system, based on.^38^ ^,^ ^39^ Briefly, the catheters were made of Silastic tubing (0.02-inch i.d. × 0.037-inch o.d.; Green Rubber, Woburn, MA) and flushed with tridodecylmethyl ammonium chloride (TDMAC) heparin (Polysciences Inc., Warrington, PA). To secure the catheter to the jugular vein, non-absorbable suture (General Medical, New Haven, CT) was used, and passed subcutaneously to exit the back through a 22-gauge cannula (Plastics One, Roanoke, VA) that was embedded in dental cement on a 1-inch Marlex surgical mesh base (Bard Inc., Cranston, RI).^39^ After the surgical procedure, animals recovered for a week before starting self-administration (SA). During surgery and one day after, mice were administered a dose of ketofen (5 mg/kg, s.c.) to reduce pain and discomfort and 2.27% enrofloxacin (0.05 ml, i.v.) to curb infections. Catheters were flushed daily with 0.2 ml of heparinized (20 IU/ml) in bacteriostatic saline solution^39^ containing Kanamycin (20 mg/ml) before each self-administration (SA) session to prevent clotting and curb infection.

### Self-administration apparatus

Operant conditioning chambers (Med Associates, East Fairfield, VT) were used for SA, extinction, and reinstatement procedures. Each chamber was located inside a ventilated, sound-attenuating cabinet that contained an injection assembly consisting of an injection pump (Razel Model A, Stamford, CT) and a 10-ml glass syringe connected to a fluid swivel (Instech, Plymouth, PA) with Teflon® tubing.^39^ Tygon® tubing was connected to the swivel and to the external port of the animal's catheter. This line was protected by a metal spring secured to Teflon® threads on the catheter assembly. Two levers (4 × 2 cm) were positioned 2 cm above the floor and assigned as active and inactive.^39^ Pressing the active lever delivered an intravenous infusion of cocaine (0.5 mg/kg over 2.5 s; 50 µl total volume). During the injection period, a cue light located above the lever and a tone were presented, while the house light was turned off. This was followed by a 12.5-s timeout period, during which the house light remained off, and further active responses had no programmed effect.^39^ The illumination of the house light indicated the end of the 15-s injection and timeout interval. Presses on the inactive lever were recorded but produced no programmed outcome.^39^

### Cocaine self-administration

Self-administration sessions began 3 h after bacterial inoculation. Mice were allowed to acquire cocaine SA (0.5 mg/kg/injection, i.v.) on a fixed-ratio 1: time-out 12.5 s reinforcement schedule in daily 2 h test sessions (or 60 infusions maximum) for 6 successive days.^39^ Catheter patency was confirmed following the final progressive ratio session and again prior to the first extinction session by administering a brief intravenous injection of sodium methohexital (0.1 mg/0.1 ml). Successful catheter function was verified by the immediate onset of short-lasting anesthesia. Animals that lost catheter patency during the experiment were excluded from further analysis.^39^

### Progressive ratio session

Two days after cocaine SA training, two cohorts of mice underwent 4 progressive ratios (PR) testing. Each PR session lasted 4 h and used 1 mg/kg/infusion cocaine on the first and second sessions, and 0.5 mg/kg on the third and fourth sessions. The number of operant responses required for each cocaine infusion increased according to the following formula: for *odd* inf.#, FR(*n*) = (*n* + 1/2)^2^; for *even* inf.#, FR(*n*) = (*n*/2)(*n*/2 + 1).^40^ The breakpoint was defined as the highest ratio of responses per injection completed before a 30-min period elapsed without earning another infusion. Data was analyzed for each dose using the average responses of the two sessions.

### Extinction sessions and reinstatement

Extinction and reinstatement sessions were performed 2 d after the cocaine SA training. To this end, two independent cohorts of mice (distinct from those used for the PR experiments) were placed in the operant chambers daily for 2 h for 5 d. During this time, the drug-paired lever was recorded, but the light and discrete tone cues were not present. 72 h after the last extinction session, animals were tested first for a cue-induced reinstatement of cocaine seeking. In this first session, the mice were placed in the SA operant chamber for 1 h of additional extinction conditions, followed by five noncontingent presentations of the drug-associated cue light and tone (light/tone for 2.5 s) every 2 min for 10 min as a primer, after which reinstatement of drug-paired lever responses was measured for an additional 1 h period.^38^ The second reinstatement session, 24 h after the first one, consisted of 1 h of additional extinction conditions, followed by an experimenter-delivered intraperitoneal cocaine injection (10 mg/kg) and 1 h of measures of the drug-paired lever responses.^38^

### Drugs

Cocaine hydrochloride was obtained from the National Institute on Drug Abuse (NIDA, Research Triangle Park), dissolved in 0.9% sterile saline, and filtered.

### Bacterial strains and growth conditions

All *E. coli* HS strains were grown overnight in Luria–Bertani (LB) broth at 250 rpm and 37 °C prior to subculture for mouse inoculation. All bacteria in the stationary phase were kept as stocks at −80 °C mixed with sterile glycerol (25% final concentration). The *E. coli* HS WT isogenic kanamycin-resistant (Km^R^) and the Δ*CycA* Km^R^ mutant strain were generated using the *λ* red recombinase method as previously described.^41^

### Bacterial inoculation and maintenance

24 h before inoculation and for the length of the cocaine SA period, mice were intravenously (i.v.) administered kanamycin (10 mg/kg) once a day to maintain bacterial colonization. For bacterial inoculation, mice were orally gavaged with 10^9^ CFU of *E. coli* HS WT or with 1 × 10^10^ CFU of *E. coli* HS Δ*CycA*. Fecal pellets were collected starting from day 1 after inoculation to quantify bacterial loads through quantitative culture on selective media (MacConkey^Km^) in aerobic conditions. On euthanasia day, cecal contents were harvested to quantify *E. coli* HS colonization.

### Glycine supplementation and levels determination

Starting at the first sessions and for the length of the cocaine SA period, mice were intravenously (i.v.) administered Glycine (300 mg/kg) once a day, right before each SA session.

Glycine levels in colon contents, serum, and NAc were determined using the Glycine Assay Kit (Cell Biolabs, Inc, cat#MET-5070, USA) as per the manufacturer’s protocol. NAc glycine concentration was normalized to the total protein levels.

### 16S rRNA sequencing and analysis

Fecal samples were collected immediately after completion of the progressive ratio task, flash-frozen, and stored at −80 °C until processing. Samples were shipped on dry ice to SeqCenter (Pittsburgh, PA) for DNA extraction, library preparation, and 16S rRNA gene sequencing. Genomic DNA was extracted using the ZymoBIOMICS DNA Miniprep Kit, quantified by Qubit fluorometry, and the V3–V4 region of the 16S rRNA gene was amplified and sequenced. Sequence processing was performed in QIIME2 (v2023.9), including primer trimming (cutadapt), denoising and ASV inference (DADA2), and taxonomic assignment using the SILVA 138 99% reference database. Downstream analyses were conducted on relative abundance tables and diversity metrics.

### Nucleus accumbens dissection and protein lysate preparation

After euthanasia, mice brains were rapidly removed, snap frozen, and stored at –80 °C until processing. For tissue dissection, bilateral punches of the NAc, predominantly the core subregion, were excised from 1-mm-thick coronal slices starting from sections corresponding to Plate 55 (−2.92 mm, anterior/posterior relative to Bregma) of Paxinos and Franklin^42^ as before.^43^ NAc tissue was then lysed in RIPA Lysis Buffer (100 mM NaCl; 50 mM Tris, pH 7.5; 1 mM EDTA; 0.5% DOC; 0.1% SDS; 1% Triton 100×) supplemented with a protease inhibitor cocktail (RPI, Cat#P50600-1). Cellular debris was pelleted by centrifugation, and supernatant, containing soluble proteins (lysate), was collected. Protein concentration was determined by Bradford assay. 10 μg of total proteins were submitted to the Rutgers Center for Advanced Proteomics Research (CAPR) were protein reduction was performed with 5 mM DTT for 30 min at 60 °C, alkylation with 20 mM iodoacetamide for an hour at room temperature in the dark and followed by SP3 beads digestion method described in Hughes et al.^44^, with trypsin (sequencing grade, Thermo Scientific Cat#90058) in 100 mM ammonium bicarbonate, 2 mM CaCl_2_ and incubated at 37 °C overnight. Peptides were acidified with formic acid, and 10% of the sample was analyzed by Liquid chromatography-tandem mass spectrometry (LC–MSMS).

### Liquid chromatography–tandem mass spectrometry (LC–MSMS)

Samples were analyzed by LC–MS using Nano LC–MSMS (Dionex Ultimate 3000 RLSCnano System, Thermofisher) interfaced with Eclipse (Thermofisher). Samples were loaded onto a fused silica trap column Acclaim PepMap 100, 75 µm×2 cm (ThermoFisher). After washing for 5 min at 5 µl/min with 0.1% TFA, the trap column was brought in-line with an analytical column (Nanoease MZ peptide BEH C18, 130A, 1.7 µm, 75 µm ×250 mm, Waters) for LC–MS/MS. Peptides were fractionated at 300 nL/min using a segmented linear gradient 4%–15% B in 30 min (where A: 0.2% formic acid, and B: 0.16% formic acid, 80% acetonitrile), 15%–25% B in 40 min, 25%–50%B in 44 min, and 50%–90%B in 11 min. Solution B then returns at 4% for 5 min for the next run.

Data independent acquisition (DIA) workflow was used to analyze the eluted peptides. MS scan range was set at 400–1200, resolution 12,000 with AGC set at 3E6 and ion time set as auto. An 8 m/z window was set to sequentially isolate (AGC 4E5 and ion time set at auto) and fragment the ions in the C-trap with a relative collision energy of 30. The MSMS were recorded with a resolution of 30,000. Raw data were analyzed with a predicted library from the Uniprot mouse reference proteome for library-free search using DIA NN 1.8.1^45^ ^,^ ^46^ with recommended settings. The results were filtered for both PEP (an estimate of the posterior error probability for the precursor identification, based on scoring with neural networks) filter < 0.01 and PG.Q (Protein Group *Q* Value) filter < 0.01. Protein group MaxLFQ values were used for quantification.

For statistical analysis, protein groups were filtered to require a minimum valid value of maxLFQ to be three for at least one of the groups to be compared. Data were analyzed using an in-house R program that modifies the package Quasiseq^47^ for analysis of mass spectrometry data.^48^ Instead of independently estimating a variance term for each protein,^49^ this method (QuasiSpectral version 0.22, https://github.com/mooredf22/quasispectral) uses a Bayesian procedure to shrink these variance term estimates toward the common mean. This improves the estimate of the variance term by drawing on information from the estimates of all the proteins. Output is provided in terms of false-discovery based q-values calculated using the original method of Benjamini and Hochberg.^49^ ^,^ ^50^

### Proteomic analysis

Identified protein names were converted to gene names for subsequent analysis. To visualize changes in protein abundance among experimental groups, volcano plots were generated using the free online platform for data visualization and graphing SRplot.^51^ Volcano plots show only proteins identified in both conditions. Proteins uniquely identified in a single condition are listed in Supplementary Table 1. Decreased proteins are shown in blue, and increased proteins are displayed in red for each condition. The x-axis represented log2(FC), indicating the magnitude of protein abundance change, and the y-axis represented the −log10(*p* value), indicating the statistical significance of the protein regulation. Proteins were identified as differentially regulated if they surpassed the predetermined thresholds for statistical significance (*p* < 0.05; –log10 *p* > 1.3) and biological relevance (fold change, FC) (|log2(FC)| ≥ 0.58, corresponding to a minimum fold change of 1.5). Gene ontology (GO) analysis was conducted using the Database for Annotation, Visualization and Integrated Discovery (DAVID, v6.8)^52^ ^,^ ^53^ based on the *Mus musculus* genome. The analysis included a Fisher's exact test followed by an FDR multiple testing correction. Pathway enrichment analysis was performed using the Kyoto Encyclopedia of Genes and Genomes (KEGG) database within DAVID, considering pathways with a *p* < 0.05 as statistically significant. To predict protein-protein interactions, the identified proteins, converted to gene names, were analyzed using the STRING software.^54^ The analysis was conducted by setting the species to *M. musculus* with a medium confidence level (score 0.4). We retrieved interactions based on experimental evidence and database annotations, excluding all other prediction methods available in STRING. Correlations between single DEPs and the Breakpoint of cocaine injection achieved for the mice during the PR task were performed using *Pearson* analysis (Supplementary File 1). Addiction-related proteins were further identified from the overall comparisons using the KEGG Cocaine Addiction pathway.

### Rank–rank hypergeometric overlap (RRHO2) analysis

To compare the proteomic changes between experimental conditions in an unbiased and threshold-free manner, we performed a Rank–Rank Hypergeometric Overlap (RRHO2) analysis using the RRHO2 R package.^55^ ^,^ ^56^ Protein lists derived from differential expression analyses were ranked by a signed metric: –log10(*p*-value) multiplied by the sign of the log2 fold change, positioning upregulated proteins at the top and downregulated ones at the bottom. Only proteins present in both datasets were retained for comparison. Ranked lists were then input into RRHO2_initialize, and the significance of overlap was computed across all rank thresholds using a hypergeometric test, producing a 2D matrix of –log10(*p*-values). Heatmaps were generated using the RRHO2_heatmap function, where regions of significant overlap indicate concordant or discordant patterns of regulation. In this output, the upper right quadrant corresponds to proteins downregulated in both conditions, the lower left quadrant to proteins upregulated in both, while discordant regulation appears in the upper left (down in list 2, up in list 1) and lower right (up in list 2, down in list 1) quadrants.

### Statistical analysis

All data are presented as means ± standard error of the mean ± S.E.M. Statistical significance was defined as *p* < 0.05. Comparisons between two experimental groups were performed using two-tailed Student’s *t*-tests, whereas relationships between variables were assessed with *Pearson*’s correlation coefficients using one or two-tailed analysis as specified in the results section. The data met the assumptions of normality and homogeneity of variance. When more than two groups were compared, one-way or two-way ANOVA was applied, followed by Bonferroni’s post hoc correction for multiple comparisons. Effect size was also calculated for comparisons between two experimental groups (*Cohen’s d*) and reported in the corresponding result section. Effect sizes were interpreted according to conventional benchmarks, where *d* < 0.2 indicates a small effect, 0.2 < *d* < 0.5 a medium effect, and *d* ≥ 0.8 a large effect.

## Results

### *Proteobacteria-*induced glycine uptake modulates host cocaine self-administration acquisition

**Figure 1.**
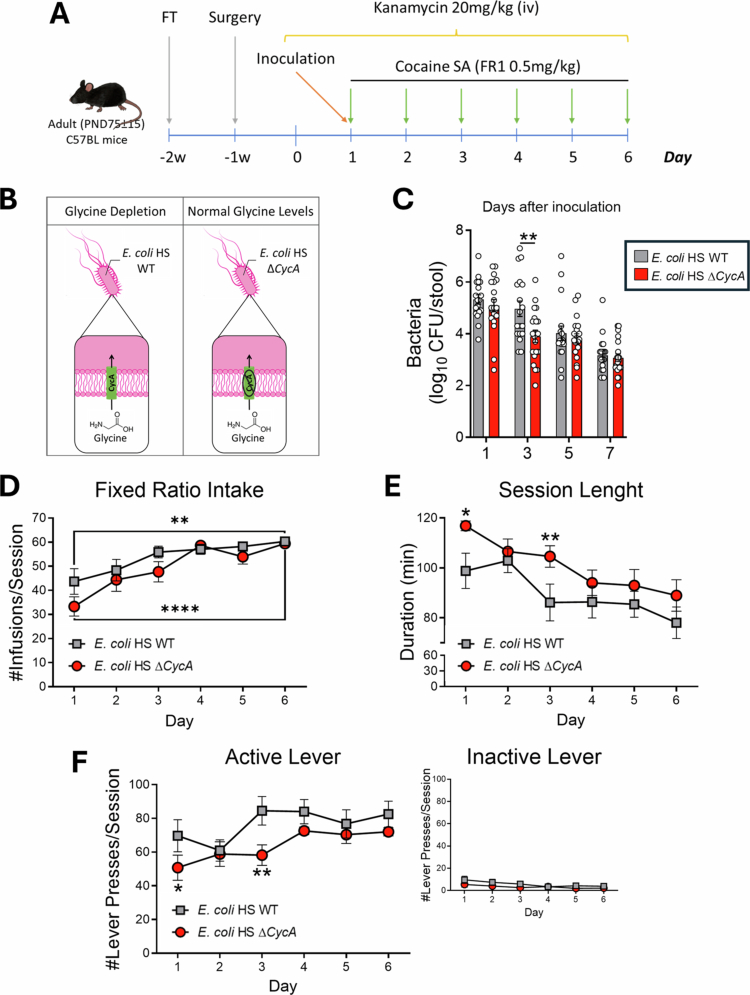
*Proteobacteria* glycine uptake modulates motivation during host cocaine self-administration (SA) acquisition. (A) Timeline and experimental procedures. (B) Schematic representation of the differences between the *E. coli* HS strains used in the study. (C) *E. coli* HS loads in colon content at 1, 3, 5, and 7 d after inoculation during SA acquisition. (D) Number of infusions received during each SA session. In comparison with animals colonized with *E. coli* HS Δ*CycA,* mice colonized with *E. coli* HS WT show, on average, shorter session durations (E) and a higher number of active lever presses (F) during the SA sessions. No differences were observed in the inactive level presses numbers (*inset*). Data presented as average ± SEM. *n *=* *19–20/4 cohorts per group. *p < 0.05 (for further details, see text).

Recently, using mouse models, we found that cocaine exposure elevates gut norepinephrine, promoting *Proteobacteria* colonization. This shift depletes host glycine, which in turn enhances cocaine-induced sensitization and conditioned place preference.^16^ Based on this data, we decided to test whether this same microbial alteration can modify cocaine SA acquisition. To this end, after surgery recovery, kanamycin-exposed adult male C57BL/6 mice were inoculated with *E. coli* HS WT or *E. coli* HS Δ*CycA* and trained to self-administer cocaine for 6 d (Figure 1A and B). Under this antibiotic treatment, stable bacterial colonization was observed for both strains in stool collected after 1,3, 5, and 7 d after inoculation (Figure 1C). Importantly, at Day 3, a significantly higher colonization in the *E. coli* HS WT strain is observed compared to the glycine-uptake–deficient mutant *E. coli* HS Δ*CycA*, (Figure 1C; two-way ANOVA for repeated measures: main effect of time F_(2.465, 88.75)_ = 38.86, *p *< 0.0001; main effect of strain F_(1, 36)_ = 4.228, *p *=* *0.0471; post hoc Bonferroni; significant differences between bacteria colonization at day 3 between *E. coli* HS WT and *E. coli* HS Δ*CycA*, *p *=* *0.0081) suggesting that the ability to deplete glycine confers a competitive colonization fitness advantage to the WT strain. No differences in colonization were observed at Days 5 and 7, consistent with the strains' colonization stabilizing.

During the cocaine SA acquisition training, intake in both groups gradually increased over the 6 training sessions (Figure 1D; two-way ANOVA for repeated measures: main effect of time F_(5, 180)_ = 14.88, *p* < 0.0001; post hoc Bonferroni; significant differences between the amount of infusions/session during day 1 vs day 6 for both groups; *E. coli* HS WT *p* = 0.0016; *E. coli* HS Δ*CycA* *p* < 0.0001). No significant differences in the number of infusions between strains or time × strains interactions were observed during acquisition training (Supplementary Figure 1A and B). However, we found that while both groups of animals presented a significant reduction in the total session length, this effect was more pronounced in the animals colonized with the WT, glycine-depleting *Proteobacteria* (Figure 1E; two-way ANOVA for repeated measures: main effect of time F_(5, 180)_ = 7.855, *p* < 0.0001; main effect of strain F_(1, 36)_ = 4.777, *p* = 0.035; post hoc Bonferroni; significant differences between the session length during day 1 vs day 6 for both groups; *E. coli* HS WT *p *=* *0.046; *E. coli* HS Δ*CycA p *=* *0.0005; and significant differences between strains in session length at day 1 *p *=* *0.025, and day 3 *p *=* *0.022). Similarly, while no differences were observed between the number of inactive lever presses between groups (Figure 1F inset; two-way ANOVA for repeated measures: main effect of time F_(5, 175)_ = 2.783, *p *=* *0.019), the mice colonized with *E. coli* HS WT showed more active lever presses during the training than the mice inoculated with *E. coli* HS Δ*CycA* (Figure 1F; two-way ANOVA for repeated measures: main effect of time F_(5, 180)_ = 3.818, *p *=* *0.0026; main effect of strain F_(1, 36)_ = 4.789, *p *=* *0.035; post hoc Bonferroni; significant differences between strains in lever presses at day 1 *p *=* *0.049, and day 3 *p *=* *0.0065). These data suggest that animals inoculated with the WT, glycine-depleting *E. coli* HS, show an increase in the operant responses associated with the acquisition of cocaine SA.

### *Proteobacteria*-induced glycine reduction increases the motivation for cocaine

**Figure 2.**
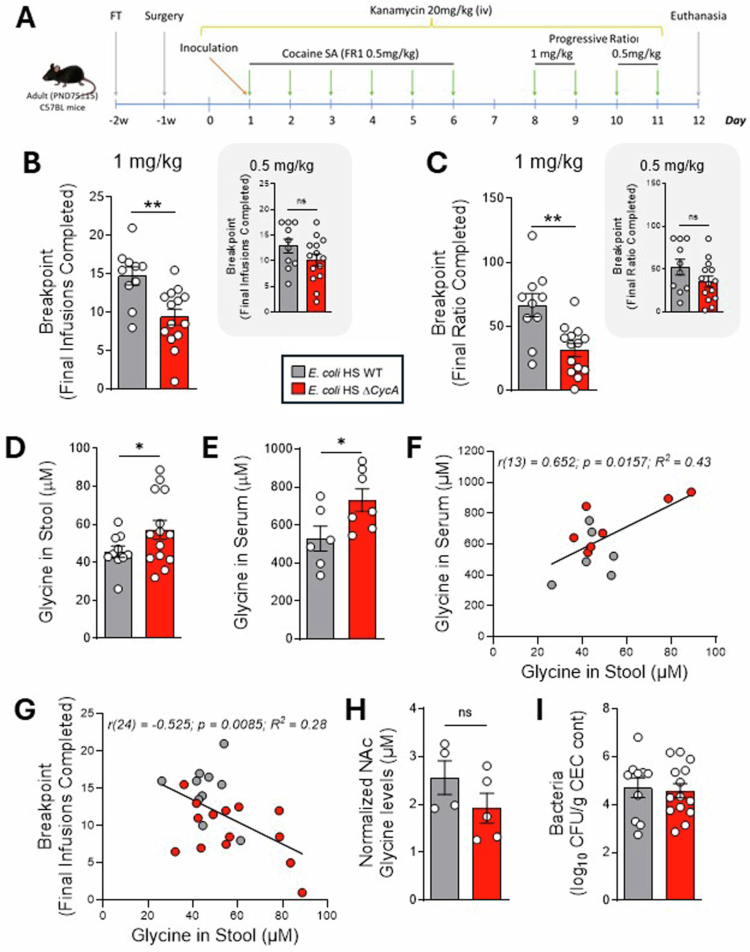
*Proteobacteria*-Induced Glycine Reduction Increases the Motivation for Cocaine. (A) Timeline of treatment and experimental procedures. Final infusion (B) and ratio (C) completed by mice colonized with *E. coli* HS WT and Δ*CycA*in the 1 mg/kg dose progressive ratio test. The data shows the average breakpoint between sessions 1 and 2. No significant differences were observed at the 0.5 mg/kg dose (B and C *insets*). Glycine levels in stool (D) and serum (E) after euthanasia, 24 h after the last progressive ration session. Glycine levels in stool and serum are positively correlated in the evaluated mice (F). Glycine stool levels and final infusion completed are negatively correlated in all the tested mice (G). NAc glycine levels (H) and *E. coli* HS loads in cecal contents (I). Data presented as average ± SEM. *n *=* *4–14/2 cohorts per group. *ns*: non-significant, *p < 0.05; **p < 0.01 (for further details, see text).

To further confirm the effects of the two different *E. coli* HS strains on the effort mice exert to maintain cocaine SA behavior, we used a progressive ratio (PR) reinforcement schedule at two cocaine injection doses (0.5 and 1 mg/kg) (Figure 2A). We found that the colonization with an *E. coli* HS WT strain significantly increases the motivation only for the 1 mg/kg of cocaine when compared with mice colonized with *E. coli* HS Δ*CycA*, as indicated by a significant difference in PR breakpoints, i.e., the highest ratio of cocaine injection (Figure 2B: *Main panel:* 1 mg/kg of cocaine, *t* test: *t*_(22)_ = 3.443, *p *=* *0.0023; Cohen’s *d *=* *1.5; inset: 0.5 mg of cocaine, *t* test: *t*_(22)_=1.500, *p *=* *0.1477; Cohen’s *d *=* 0.6*) or lever-presses achieved (Figure 2C: Main panel: 1 mg/kg of cocaine, *t* test: *t*_(22)_=3.641, *p* = 0.0014; Cohen’s *d* = 1.5; inset: 0.5 mg of cocaine, *t* test: *t*_(22)_=1.579, *p* = 0.1286; Cohen’s *d* = 0.7) before animals voluntarily cease self-administration behavior.

After the last PR test, mice were euthanized (Figure 2A). Glycine was determined in stool samples, confirming that *E. coli* HS WT inoculated mice have lower glycine levels than mice with *E. coli* HS Δ*CycA* (Figure 2D, Unpaired *t* test with Welch's correction: *t*_(20.23)_=1.99, *p* = 0.05; Cohen’s *d* = 0.9). A subset of animals was also used to measure glycine levels in serum (Figure 2E). As expected, glycine was significantly lower in the animals colonized with the *E. coli* HS WT strain than in those colonized with the *E. coli* HS Δ*CycA* mutant, supporting the idea that microbiota-driven glycine depletion has systemic consequences (*t* test: *t*_(11)_=2.282, *p* = 0.043; Cohen’s *d* = 1.4). Consistent with this, glycine levels in stool positively correlate with serum glycine levels (Figure 2F), further reinforcing the link between local microbial metabolism and systemic glycine availability. Glycine levels in the stool of the evaluated animals were negatively correlated with the highest ratio of cocaine injection achieved for the mice during the PR task (Figure 2G). Intriguingly, at the time of euthanasia, glycine levels in the NAc were not different between animals colonized with the *E. coli* HS WT strain and those colonized with the *E. coli* HS Δ*CycA* mutant (Figure 2H). Therefore, to validate the role of glycine depletion in cocaine-associated responses, we perform a rescue experiment by exogenously supplementing this amino acid to mice colonized with *E. coli* HS WT (Supplementary Figure 2A). We found that glycine administration did not significantly alter SA intake (Supplementary Figure 1A and B) or acquisition (Supplementary Figure 2B–E). However, this supplementation was sufficient to prevent the bacterial-induced increase in the PR breakpoint (Supplementary Figure 2J and K), further supporting that *Proteobacteria*-dependent glycine depletion impacts the motivation associated with cocaine SA. In line with the previous observations (Figure 2D and H), while glycine levels in stools were significantly altered (Supplementary Figure 2H), no differences were observed in the NAc among groups (Supplementary Figure 2I). At euthanasia, bacterial loads in cecal contents were measured, showing that both strains had similar colonization levels (Figure 2I, *t* test: *t*_(22)_=0.2556, *p *=* *0.80; Cohen’s *d *=* *0.1 and Supplementary Figure 2G).

Finally, to corroborate that the introduction of the two different *E. coli* strains did not substantially alter the overall microbiome structure, 16S rRNA sequencing was performed in stool samples collected at euthanasia. No significant differences were observed (Supplementary Figure 3).

Taken together, these data show that *Proteobacteria*-induced glycine depletion increases motivation for cocaine.

### *Proteobacteria*-induced glycine reduction differentially alters cocaine-induced plasticity in the nucleus accumbens after progressive ratio

**Figure 3.**
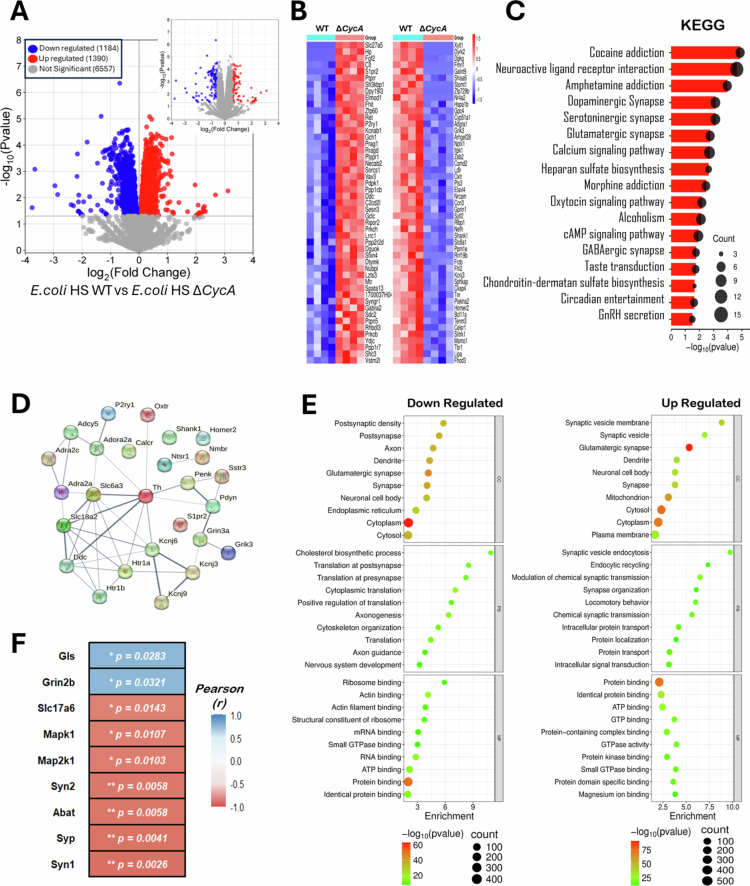
Proteomic alterations in the nucleus accumbens after progressive ratio in mice colonized with the *E. Coli* HS WT or the Glycine Transporter Deficient Mutant *E. coli* HS Δ*CycA.* (A) Volcano plot of differentially expressed proteins in the Nucleus Accumbens in *E. coli* HS WT T in comparison with *E. coli* HS Δ*CycA* 24 h after the last testing session. Red dots indicate significantly upregulated proteins and blue dots downregulated proteins (*p *<* *0.05). *Insert*: differentially expressed proteins (DEPs) with fold change FC ≥ 0.58. (B) Heat maps showing the top 50 most significantly upregulated or downregulated DE proteins. (C) Significantly enriched KEGG pathways analysis based on the DE proteins with FC ≥ 0.58. (D) String analysis showing the network between the proteins related to the top 6 differentially regulated KEGG pathways. Network nodes represent proteins; edges represent both functional and physical protein associations; line thickness indicates the strength of data support. (E) Gen Ontology analysis of the DE proteins. (F) Linear correlation profile between selected differentially expressed synaptic protein abundance and final infusion completed (Breakpoint) during the PR test (*Pearson* correlation analysis, *p < 0.05; **p < 0.01; one-tail *t* test; Gls: glutaminase, Grin2b: glutamate ionotropic receptor NMDA type subunit 2B, Slc17a6: Vesicular Glutamate Transporter 2 (VGLUT2); Mapk1: mitogen-activated protein kinase 1; Map2k1: mitogen-activated protein kinase 1; Syn2: Synapsin II; Abat: 4-aminobutyrate aminotransferase; Syp: Synaptophysin; and Syn1: Synapsin I). *n *= 4/2 cohorts per group (for further details, see text).

To delineate protein regulatory dynamics between the two different *E. coli* HS strains after cocaine exposure, we performed a proteomic analysis of the NAc 24 h after the PR evaluation (Figure 3). We found that out of 9,131 detected proteins in both groups, a total of 2574 were differentially expressed, with 1390 upregulated and 1184 downregulated in the *E. coli* HS Δ*CycA* inoculated mice, compared to animals inoculated with the WT strain (Figure 3A and B). Then, based on the differentially expressed proteins (DEPs), we performed pathway analysis using the Kyoto Encyclopedia of Genes and Genomes (KEGG) only in proteins that showed a FC ≥ 1.5 (Figure 3A *inset* and 3C). Not surprisingly, we found that the most impacted pathways between mice colonized with *E. coli* HS WT and *E. coli* HS Δ*CycA* after PR, were the ones associated with addiction (cocaine and amphetamine), dopaminergic synapse, and glutamatergic synapse (Figure 3C). Furthermore, and to investigate potential protein–protein interactions among these DEPs, we performed a functional network analysis using STRING, including the proteins identified in the top 6 most altered pathways (Figure 3D). This analysis revealed a highly connected module involving key neurotransmitter pathways and neurotransmitter receptors, including dopamine, glutamate, and opioid peptides. Then, we evaluated differences in biological processes, molecular functions, and cellular components using the Gene Ontology (GO) enrichment analysis (Figure 3E). To better capture potentially subtle, yet biologically relevant changes in protein expression, we included all the identified DEPs. We found several key neurobiological processes differentially represented between *E. coli* HS WT and *E. coli* HS Δ*CycA* colonized mice, from pathways related to synaptic function, neurotransmitter signaling, to different binding processes. Finally, we performed a correlational analysis between the abundance of DEPs selected based on the KEGG Cocaine Addiction pathway and the cocaine Breakpoint observed in the PR task. Using this analysis, we found crucial single proteins,^57-64^ including synaptic, glutamatergic, and GABAergic signaling components, that were highly correlated with the behavioral outcomes (Figure 3F). Altogether, these data indicate that gut colonization with the WT glycine-depleting *E. coli* HS strain interferes with NAc neuroadaptations associated with cocaine consumption, thereby increasing drug-associated motivational behaviors.

### *Proteobacteria*-induced glycine reduction alters drug-seeking behaviors

**Figure 4.**
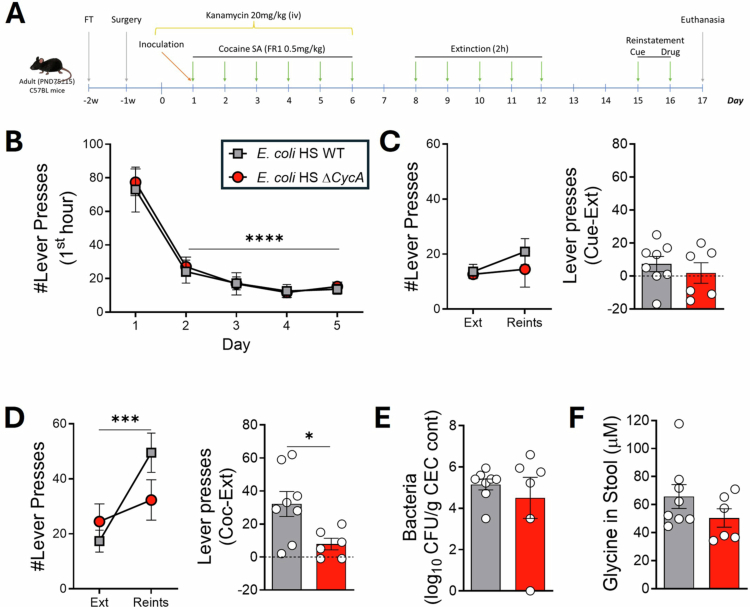
*Proteobacteria*-Induced Glycine Reduction Alters Drug-Seeking Behaviors. (A) Timeline of treatment and experimental procedures (B) All the mice significantly decreased active lever pressing from the first day of extinction to the last. (C) *Left panel:* number of lever presses during the first extinction hour and during the hour following the cue in the reinstatement session. *Right panel:* difference in the number of lever presses performed in the hour after the cue and the extinction hour. (D) *Left panel:* number of lever presses during the first extinction hour and during the hour following an i.p. cocaine injection in the reinstatement session. *Right panel:* difference in the number of lever presses performed in the hour after the i.p. cocaine injection and the extinction hour. (E) *E. coli* HS loads in cecal contents and glycine levels in stool (F) after euthanasia, 24  h after the last reinstatement evaluation. Data presented as average ± SEM. *n *=* *8–6/2 cohorts per group. *p < 0.05; **p < 0.01; ****p < 0.0001 (for further details, see text).

Since we found significant differences in the effects induced by the colonization with the different *E. coli* HS strains (WT or Δ*CycA*) on the PR responses (Figure 2) as well as on the proteomic profile of the NAc (Figure 3), we decided to test whether these alterations in the gut microbiome also led to changes in cocaine-seeking behavior after an extinction training (Figure 4). Upon initial exposure to the cocaine-paired environmental context in extinction testing, all animals exhibited strong reductions in cocaine-seeking behavior at the drug-paired lever (Figure 4B; two-way ANOVA for repeated measures: main effect of time F_(4, 48)_ = 56.22, *p* < 0.0001; post hoc Bonferroni; significant differences between number of lever presses during day 1 vs all the other days for both groups; *p < *0.001). No significant differences between groups for reinstatement elicited by cues when compared to extinction baselines (Figure 4C; left panel: two-way ANOVA for repeated measures: time × strains interactions F_(1, 12)_ = 0.5202, *p* = 0.485; effect of time F_(1, 12)_ = 1.463, *p = *0.249; effect of strain F_(1, 12)_ = 0.6080, *p* = 0.450; right panel: *t* test: *t*_(12)_=0.7213, *p* = 0.485; Cohen’s *d* = 0.4). However, after a drug-primed reinstatement, the animals colonized with *E. coli* HS WT show a significant cocaine seeking response, which was absent in *E. coli* HS Δ*CycA* colonized mice (Figure 4D; left panel: two-way ANOVA for repeated measures: main time × strains interactions F_(1, 12)_ = 6.801, *p = *0.029; main effect of time F_(1, 12)_ = 18.40, *p *=* *0.0011; post hoc Bonferroni, significant differences between number of lever presses (Coc-Ext) between strains *p* = 0.0004; right panel: *t* test: *t*_(12)_=2.608, *p* = 0.0229; Cohen’s *d* = 1.5). 24 h after the last behavioral evaluation, mice were euthanized (Figure 4A). Bacterial loads in the cecum content and glycine levels in colon contents were measured. At this time point, both strains were still present (Figure 4E, Unpaired *t* test with Welch's correction: *t*_(5.715)_=0.6308, *p* = 0.553; Cohen’s *d* = 0.5), and glycine levels were similar between groups (Figure 4F, *t* test: *t*_(12)_=1.344, *p* = 0.203; Cohen’s *d* = 0.8). These results indicate that the initial glycine depletion induced by the *E. coli* HS WT strain not only exacerbates drug-associated motivational behaviors but also significantly alters drug-induced seeking responses after extinction.

### Convergent nucleus accumbens proteomic profiles in mice colonized with *E. coli* HS WT and *E. coli* HS Δ*CycA* after reinstatement

**Figure 5.**
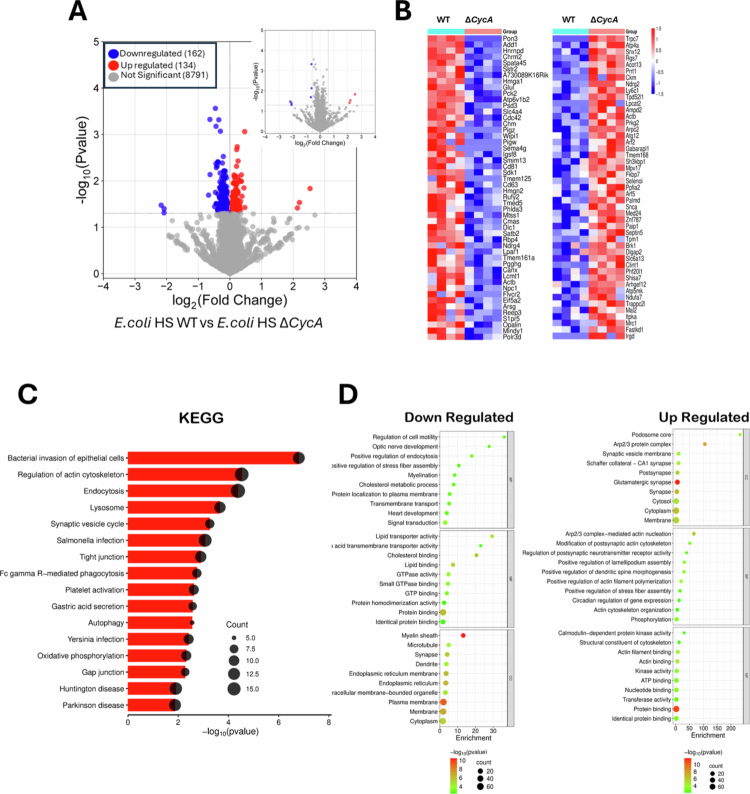
Proteomic Alterations in the Nucleus Accumbens after the Reinstatement in Mice Colonized with the *E. coli* HS WT or the Glycine-uptake-Deficient mutant *E. coli* HS Δ*CycA.* (A) Volcano plot of differentially expressed proteins in the Nucleus Accumbens in *E. coli* HS WT T in comparison with *E. coli* HS Δ*CycA* 24 h after the last testing session. Red dots indicate significantly upregulated proteins and blue dots downregulated proteins (*p *<* *0.05). *Insert*: Differentially Expressed Proteins (DEP) with Fold change FC ≥ 0.58. (B) Heat maps showing the top 50 most significantly upregulated or downregulated Differentially Expressed Proteins (DEPs). (C) Significantly enriched KEGG pathways analysis based on the DEPs, *p* ≤ 0.05. (D) Gen Ontology analysis of the DEPs. *n *=* *4/2 cohorts per group (for further details, see text).

After evaluating the reinstatement responses, we performed a proteomic analysis of the NAc to delineate protein regulatory dynamics between the mice colonized with the different *E. coli* HS strains (Figure 5). Different proteomic alterations were observed between the two groups (Figure 5A and B). However, these changes appeared to be more subtle compared to the PR phase, with fewer proteins showing significant up- or down-regulation (Figure 5A, *inset*). Given the smaller number of proteins showing a FC ≥ 1.5 (Figure 5A; *inset*), we opted to include all the DEPs to perform KEGG (Figure 5C) and GO (Figure 5D) enrichment analyses. In contrast to the PR phase, in these analyses, we found mostly pathways associated with host-microbiota interaction and metabolic and metabolomic processes.

### The proteomic profile of the nucleus accumbens follows a different trajectory between mice colonized with *E. coli* HS WT and *E. coli* HS Δ*CycA*

**Figure 6.**
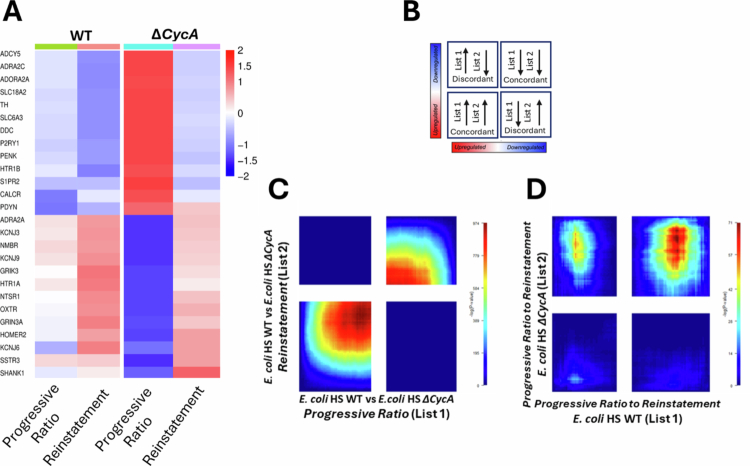
The Proteomic Profile of the Nucleus Accumbens Follows a Different Trajectory between Mice Colonized with *E. coli* HS WT and *E. coli* HS Δ*CycA.* (A) Heat maps showing the progression of proteins associated with the top six most altered pathways identified after the progressive ratio between mice colonized with the *E. coli* HS WT or the glycine-uptake-deficient mutant Δ*CycA*. Rank–rank hypergeometric overlap (RRHO) plots allow for threshold-free comparisons in proteomic expression between two differential expression lists (B). Progression in proteomic expression between the *E. coli* HS WT and *E. coli* HS Δ*CycA* during the progressive ratio and reinstatement (C). Progression in proteomic expression from the progressive ratio to reinstatement in mice colonized with *E. coli* HS WT and *E. coli* HS Δ*CycA* (D). *n *=* *4/2 cohorts per group (for further details, see text).

Since fewer changes in the NAc were observed after reinstatement between the different colonization conditions, we decided to focus on evaluating the progression between the two time points evaluated. We started by testing the progression of proteins associated with the top 6 most altered pathways we identified after cocaine PR. We noticed a clear pattern of changes in these genes, with a marked transition between PR and reinstatement in mice colonized with *E. coli* HS Δ*CycA*, in contrast to a more subtle transition in the *E. coli* HS WT animals (Figure 6A). Based on this initial observation, we moved to a more complex and large-scale evaluation of concordance and discordance and performed a rank–rank hypergeometric overlap (RRHO2) analysis. This approach enables threshold-free, proteome-wide comparison of two ranked differential expression lists, allowing for the assessment of global similarities, differences, and directional relationships between conditions (Figure 6B). In our analysis, protein lists were ranked based on a composite score combining statistical significance and effect size (–log10 *p*-value × sign of log2 FC), thus preserving information from both magnitude and reliability of the observed changes. The RRHO2 map revealed a strong overlap of up- and down-regulated proteins between the PR and reinstatement phases (Figure 6C), suggesting consistent directional regulation of specific protein sets in *E. coli* HS WT and *E. coli* HS Δ*CycA* colonized mice across different stages of cocaine exposure, even when some of these proteins do not reach statistical significance. Then, we compared how the proteomic profile of the NAc from the two different groups of mice transitioned from PR to reinstatement (Figure 6D). The resulting heatmaps revealed a marked asymmetry between groups and a prominent hotspot in the upper right quadrant, indicating strong concordance in proteins downregulated in both genotypes, with an effect more pronounced in *E. coli* HS Δ*CycA* colonized animals. Notably, a second hotspot appeared in the upper left quadrant, representing a subset of proteins upregulated in mice colonized with the *E. coli* HS WT strain and downregulated in the *E. coli* HS Δ*CycA* group, suggesting a directional discordance. A weaker signal was also observed in the lower left quadrant, reflecting concordant upregulation, and in the lower right quadrant, indicating a small subset of proteins downregulated in mice colonized with the *E. coli* HS WT strain and upregulated in the *E. coli* HS Δ*CycA*. Together, these results indicate that specific alterations in gut *Proteobacteria*'s ability to use glycine during cocaine SA can significantly impact drug-induced brain plasticity in the host.

## Discussion

The gut–microbiota–brain axis has emerged as a key modulator in different psychiatric contexts, including SUDs, with clinical evidence showing alterations in the overall gut microbiome composition of patients^30^ ^,^ ^32^ ^,^ ^65^ ^,^ ^66^ ^,^ ^67-69^ and preclinical data supporting a contribution of gut bacteria in drug-induced neurobehavioral plasticity.^13^ ^,^ ^15^ ^,^ ^16^ ^,^ ^18^ ^,^ ^23-28^ ^,^ ^70^ Working in mice, we recently demonstrated that a bloom in gut *Proteobacteria* abundance can deplete glycine for the host, altering cocaine-induced plasticity at the level of the NAc transcriptome and enhancing behavioral sensitization and conditioned place preference.^16^ Here, we expanded upon these findings by showing that the gut *Proteobacteria* colonization also modulates more complex and translational addiction-like behaviors associated with cocaine SA. Using a genetically modified *E. coli* HS strain (*E. coli* HS Δ*CycA*), we demonstrated that glycine usage by gut *Proteobacteria* affects not only the motivation of the mice to obtain the drug but also cocaine-induced reinstatement after extinction. At the level of the brain, we showed that these behavioral alterations are accompanied by signature changes in the proteomic landscape of the NAc, providing new mechanistic insight into how the gut microbiome influences CUDs.

Although sparse, some evidence has shown that antagonists targeting specifically the glycine modulatory site of NMDA receptors did not alter cocaine self-administration.^71^ In line with this evidence, our data shows that neither of the *E. coli* strains used for mouse inoculation significantly affected the drug intake during cocaine SA. However, we found that mice colonized with the glycine-depleting *E. coli* HS WT strain exhibited shorter session lengths and higher numbers of active lever presses during SA training than those colonized with *E. coli* HS Δ*CycA*, suggesting an increase in cocaine operant responses.^72^ Although this difference was observed throughout the entire acquisition phase, it is more pronounced on Days 1 and 3. Analysis of bacterial loads 24 h after Day 1 revealed no significant differences between *E. coli* HS WT and *E. coli* HS Δ*CycA* colonization, suggesting that the behavioral differences observed on Day 1 would not be associated with changes in colonization *per se*, and may reflect acute effects related to gavage administration, transient inflammatory responses, or other short-term physiological changes during this early time point. Interestingly, 24 h after Day 3, we found significantly greater colonization in animals inoculated with the *E. coli* HS WT strain. This finding indicates that, in the context of cocaine exposure and during this period of colonization, the ability to use glycine confers a competitive fitness advantage to the WT strain, a result validated by exogenous glycine supplementation in mice. While we cannot completely exclude host immune activation as a contributor to some of the behavioral modifications observed, it is unlikely to occur in this context, since *E. coli* HS is a well-characterized commensal, non-pathogenic strain.^73^ Furthermore, prior work from our group has shown that colonization with this bacterium does not induce overt inflammation, sickness-like behavior, or systemic immune activation.^16^ Altogether, these data indicate that *Proteobacteria*-mediated glycine depletion is the major driver of the behavioral alterations observed during this task.

In line with the effects observed in operant responses during cocaine SA acquisition, our data revealed that mice colonized with the glycine-depleting *E. coli* HS WT show greater motivation to obtain the drug, with higher breakpoints in the progressive ratio test, than those colonized with the *E. coli* HS Δ*CycA* strain. Importantly, exogenous glycine administration prevents the increase in the PR breakpoint in animals colonized with *E. coli* HS WT. Moreover, although both *E. coli* HS WT and *E. coli* HS Δ*CycA* colonized mice showed comparable extinction responses, only animals colonized with the *E. coli* HS WT strain exhibited a significant cocaine-induced reinstatement, supporting a long-lasting effect, even after extinction training. It is worth noting that extinction learning and reinstatement are supported by partially dissociable neural circuits^74-77^ and dissociation between extinction performance and reinstatement has been previously reported.^78^ ^,^ ^79^

We have previously shown that glycine levels in the gut match circulating and cerebrospinal fluid glycine concentrations.^80^ In the present study, we found a similar depletion in colon contents and serum associated with bacterial glycine utilization, which can decrease central glycine availability. However, our NAc data, collected several days after the end of treatment, suggest that this central decrease would be transient and would trigger compensatory adaptations in the brain. Supporting this, previous studies have shown that central amino acid homeostasis can become uncoupled from peripheral amino acid availability through compensatory metabolic regulation.^81^ ^,^ ^82^ Similarly, systemic sensors, including intestinal neuropod cells and hepato-portal systems, can signal the brain via the vagus nerve to respond to systemic metabolic alterations.^83^ ^,^ ^84^ In line with this idea, mice supplemented with exogenous glycine, while showing elevated amino acid levels in stool, displayed glycine levels in the NAc similar to those of untreated *E. coli* HS-colonized animals.

Different NMDA receptor modulators have been previously explored using pharmacological strategies in the context of cocaine motivation and reinforcement, leading to different results.^71^ ^,^ ^85^ ^,^ ^86^ However, how the alterations of glycine or the specific modulation of the glycine-binding site influence motivation or relapse-like behavior after cocaine SA remains less explored. Among the available evidence, it has been shown that the systemic administration of D-serine, a co-agonist at the NMDA receptor glycine-binding site, during the extinction training, significantly reduces cocaine-primed reinstatement.^85^ In the same line, pharmacological strategies aimed at increasing glycine availability have also been shown to reduce cocaine-related behaviors. Specifically, selective inhibition of the glycine transporter-1, which is expressed on glial cells and postsynaptic glutamatergic neurons near NMDA receptors,^87^ ^,^ ^88^ facilitated cocaine-cue extinction learning and attenuated the reacquisition of cocaine-seeking behavior.^89^ Beyond glycine's role as an NMDA receptor co-agonist, glycine receptors (GlyRs) are also widely expressed in brain regions involved in reward and addiction, including the NAc. Although direct evidence for the role of glycine and GlyRs in cocaine addiction is limited,^16^ extensive work in alcohol research has established that GlyRs are critical molecular targets of ethanol, where their activation enhances inhibitory neurotransmission and contributes to alcohol’s reinforcing properties, particularly in the NAc.^90-92^ Therefore, a modulatory role of these receptors in the neuroadaptations associated with psychostimulant use cannot be ruled out, where they could participate in shaping excitatory–inhibitory balance within the NAc.

The NAc represents a key node in substance use disorders. In line with a modulatory role of glycine depletion on addiction, our proteomic analysis revealed a distinct signature between mice colonized with *E. coli* HS WT and animals colonized with the glycine-depletion-deficient strain *E. coli* HS Δ*CycA* after cocaine SA. KEGG pathway and Gene Ontology analyses consistently revealed that, across the two colonization groups, the most strongly regulated processes were addiction, dopaminergic, and glutamatergic synapses. STRING-based network analysis revealed a highly connected module centered around tyrosine hydroxylase (TH), with strong links to dopamine synthesis pathways (Ddc, Slc6a3, Slc18a2), glutamatergic signaling (Grin3a, Grik3), and neuropeptide systems (Penk, Pdyn). Furthermore, correlational analysis identified significant associations between synaptic, GABAergic, and glutamatergic proteins and motivational responses to cocaine. More specifically, we found that reductions in synaptic proteins, including Synapsin 1/2 and Synaptophysin, as well as the GABAergic regulator enzyme 4-aminobutyrate aminotransferase, are associated with higher breakpoints during PR. On the other hand, glutamatergic proteins (Glutaminase and Glutamate Ionotropic Receptor NMDA Type Subunit 2B) reveal a positive correlation with breakpoints during PR. These alterations in protein expression and their molecular pathways have been previously observed in the context of cocaine exposure.^57-64^ Altogether, these modifications align with the neurobiology of addiction, from initiation,^34^ to drug seeking and reinstatement.^34^ ^,^ ^93^ The recurrent identification of these pathways across experiments and animal models of addiction^16^ highlights glycine as a central mechanism influencing multiple stages of addiction. Accordingly, microbiome-driven alterations in glycine metabolism play a critical role in shaping host vulnerability to this disorder.

Our data show that, while animals remained colonized throughout SA, glycine reduction in colon contents was no longer observed after reinstatement. This suggests that a compensatory glycine response may be occurring in the gut at the host level, where the host might regulate luminal glycine availability through altered synthesis, transport, absorption, or metabolic flux,^94^ and such adaptive responses may counterbalance bacterial glycine utilization during prolonged bacterial colonization periods.^95^ At the same time, once established in the gut, the bacterium’s metabolic requirements usually change and do not necessarily imply constant metabolic output, as microbial gene expression and metabolic activity are highly context-dependent and influenced by host physiology and environmental conditions.^96^ Glycine depletion is observed in the early periods of SA, during which cocaine-induced neuroplasticity is actively developing, and most likely these early metabolic perturbations are sufficient to induce persistent adaptations within reward-related neural circuits.^97^ In line with this idea, proteomic variations in the NAc during reinstatement were more subtle than those detected during the PR phase, with fewer proteins reaching significant up- or down-regulation. Future experiments will explore whether, at this time point, other brain regions, particularly those involved in executive control, cue processing, and decision-making, show more pronounced changes and drive this drug-seeking behavior.^34^ ^,^ ^98^

To further compare patterns of proteomic regulation between *E. coli* HS WT and *E. coli HS* Δ*CycA* colonized mice across the two cocaine SA stages tested, we used RRHO2 analysis. This evaluation revealed a strong overlap of both up- and downregulated proteins between PR and reinstatement phases. However, the transition from PR to reinstatement within each colonization group revealed a clear divergence. Mice colonized with *E. coli* HS Δ*CycA* displayed a pronounced and coordinated proteomic shift, particularly among concordantly downregulated proteins; whereas *E. coli* HS WT colonized mice showed minimal overlap, suggesting a weaker or more heterogeneous proteomic response across these stages. We also observed these divergences by analyzing the trajectories of proteins belonging to the six most significantly altered pathways at the PR stage. This analysis revealed a marked remodeling from PR to reinstatement in *E. coli* HS Δ*CycA* colonized mice, in contrast to a more subtle and stable pattern in *E. coli* HS WT-colonized animals. In other words, in contrast to the broader and more dynamic remodeling observed in *E. coli* HS Δ*CycA* mice, *E. coli* HS WT animals show a more subtle proteomic reorganization after relapse, which could suggest an underlying increased vulnerability to drug-seeking behavior in these mice. Altogether, this data further supports that each colonization condition exerts a distinct molecular influence that shapes the neurobehavioral progression from motivational drive to relapse. These proteomics differences likely reflect variations in neural circuit engagement and synaptic plasticity within the NAc.

In summary, our findings show that the gut microbiome is a crucial factor that can modulate addiction-like behaviors. More specifically, we demonstrated that the ability of gut *Proteobacteria* to metabolize glycine during cocaine SA shapes the trajectory and long-term effects of cocaine-induced neurobehavioral plasticity, influencing the vulnerability of the NAc proteome to move from states of maintenance or structural adaptation between drug-seeking and reinstatement.

## Limitations of the study

Here, we show that the ability of gut *Proteobacteria* to use glycine during cocaine SA shapes the trajectory and long-term neurobehavioral plasticity induced by cocaine. We acknowledge that there are limitations in our studies. First, sucrose pre-training was implemented to synchronize the onset of operant self-administration with bacterial colonization. While still broadly used,^99-102^ this design choice can sometimes influence subsequent behavioral performances.^103^ Second, animals were pretreated with kanamycin, a Gram-negative–targeting, non-absorbable antibiotic. While both experimental groups received identical antibiotic treatment and no differences in overall microbiome structure (alpha or beta diversity) were observed, an indirect or untargeted effect of the antibiotic cannot be completely ruled out. Finally, we acknowledge that all these experiments were conducted in male mice and that there is potential for sex-dependent differences, particularly given known variations in both gut microbiome composition and cocaine-related behaviors associated with sex. Future experiments will focus on addressing this critical question.

## Supplementary Material

Supplementary File 1.xlsx Supplementary File 1.xlsx

STable_1.xlsx STable_1.xlsx

Supplementary Material Supplementary_File.docx

Supplementary Material Supp files.zip

## Data Availability

Proteomic and 16S rRNA sequencing data that support the findings of this study are available at https://doi.org/10.5281/zenodo.17362536 and from the corresponding author, S.C., upon reasonable request.
