# Supplementary material for: Gut Proteobacteria glycine metabolism regulates neuroplasticity, motivation, and reinstatement of cocaine self-administration in mice
Source: Gut Microbes. 2026 Jun 26;18(1):2693397. doi: 10.1080/19490976.2026.2693397 (PMC13313183; doi:10.1080/19490976.2026.2693397)
Supplement: Supplementary Material — Supplementary_File.docx [file KGMI_A_2693397_SM1620.docx]

**Supplementary Figure 1: Drug Intake is not Affected by the Different Experimental Conditions.** **(A)** Fixed ratio intake per session during the acquisition of cocaine self-administration (two-way ANOVA for repeated measures: main time × strains interactions F_(10, 215)_=1.941, *p=0.0413;* main effect of time F_(5, 215)_=9.223, *p<0.0001;* post hoc Bonferroni; significant differences between drug intake during day 1 vs day 6 for both groups; *E. coli* HS WT ***p=0.021*; *E. coli* HS Δ*CycA* *****p<0.0001;* significant differences between *E. coli* HS WT and *E. coli* HS Δ*CycA* in drug intake during day 1 *†p=0.039*). **(B)** Total cocaine intake for animals evaluated in progressive ratio (*left panel*) and extinction-reinstatement (*right panel*). Data presented as average ± SEM. *n=8–20*/2 cohorts per group. *ns*: non-significant.

**Supplementary Figure 2: Exogenous Glycine Supplementation is Sufficient to Reverse Proteobacteria-Mediated Increases in Motivation for Cocaine.** **(A)** Timeline and experimental procedures. Number of infusions received during each SA session **(B)** (two-way ANOVA for repeated measures: main time × strains interactions F_(10, 215)_=1.941, *p=0.0413;* main effect of time F_(5, 215)_=9.223, *p<0.0001;* post hoc Bonferroni; significant differences between drug intake during day 1 vs day 6 for both groups; *E. coli* HS WT ***p=0.021*; *E. coli* HS Δ*CycA* *****p<0.0001;* significant differences between *E. coli* HS WT and *E. coli* HS Δ*CycA* in drug intake during day 1 *†p=0.039)*, session durations **(C)** (two-way ANOVA for repeated measures: main effect of time F_(5, 215)_=8.937, *p<0.0001;* post hoc Bonferroni; significant differences between drug intake during day 1 vs day 6 for both groups; *E. coli* HS WT **p=0.03*; *E. coli* HS Δ*CycA* ****p=0.0002)*, active lever presses **(D)** and inactive level presses **(E)** number during the SA sessions. **(F)** *E. coli HS* loads in colon content at 1, 3, 5, and 7 days after inoculation during SA acquisition (two-way ANOVA for repeated measures: main effect of time F_(3, 126)_=1.532, *p<0.0001;* main effect of treatment F_(2, 42)_=6.581, *p=0.0033;* post hoc Bonferroni; significant differences in colonization in day 3 between *E. coli* HS WT and *E. coli* HS Δ*CycA* ***p=0.0019 and between E. coli* HS WT + Gly and *E. coli* HS Δ*CycA* **p=0.025)*. *E coli* HS loads in cecal contents **(G).** Glycine levels in stool **(H)** and NAc **(I)** (**H**: *one-way ANOVA F_(2,27)_=18.84, p<0.0001,* post hoc Bonferroni; significant differences between *E. coli* HS WT + Gly and *E. coli* HS WT and Δ*CycA;* *****p<0.0001).* Final infusion **(J)** and ratio **(K)** completed by each group of mice during the progressive ratio test (**J**: *Main panel:* 1mg/kg of cocaine*, one-way ANOVA F_(2,29)_=6.794, p=0.0038,* post hoc Bonferroni; significant differences between *E. coli* HS WT and *E. coli* HS Δ*CycA* ***p=0.0022 and between E. coli* HS WT and *E. coli* HS WT + Gly **p=0.0426; inset:* 0.5mg of cocaine, *one-way ANOVA F_(2,29)_=1.40, p=0.0258,* post hoc Bonferroni; significant differences between *E. coli* HS WT and *E. coli* HS WT + Gly **p=0.0149*) or lever-presses achieved (**K**: Main *panel:* 1mg/kg of cocaine*, one-way ANOVA F_(2,29)_=7.987, p=0.0017,* post hoc Bonferroni; significant differences between *E. coli* HS WT and *E. coli* HS Δ*CycA* ***p=0.0013 and between E. coli* HS WT and *E. coli* HS WT + Gly **p=0.0127; inset:* 0.5mg of cocaine, *one-way ANOVA F_(2,29)_=4.818, p=0.0156,* post hoc Bonferroni; significant differences between *E. coli* HS WT and *E. coli* HS WT + Gly ***p=0.0086*). The data show the average breakpoint between sessions 1 and 2. Data presented as average ± SEM. *n=8–14*/2 cohorts per group. *ns*: non-significant.

**Supplementary Figure 3: Diversity metrics are not altered between mice colonized with *E. coli* HS WT and *E. coli* HS Δ*CycA*.** No changes in alpha-diversity metrics **(A to D)** or beta-diversity when evaluated using PERMANOVA and homogeneity of dispersion (betadisper) based on Bray–Curtis and Jaccard matrices **(E)**. df: degrees of freedom, permu: number of permutations.

**Supplementary Table 1: Proteins Uniquely Identified in the Nucleus Accumbens of Mice Colonized with *E. coli* HS WT and *E. coli* HS Δ*CycA* after Progressive Ratio or Reinstatement.**
