# Supplementary figures and images for: Gut Proteobacteria glycine metabolism regulates neuroplasticity, motivation, and reinstatement of cocaine self-administration in mice
Source: Gut Microbes. 2026 Jun 26;18(1):2693397. doi: 10.1080/19490976.2026.2693397 (PMC13313183; doi:10.1080/19490976.2026.2693397)

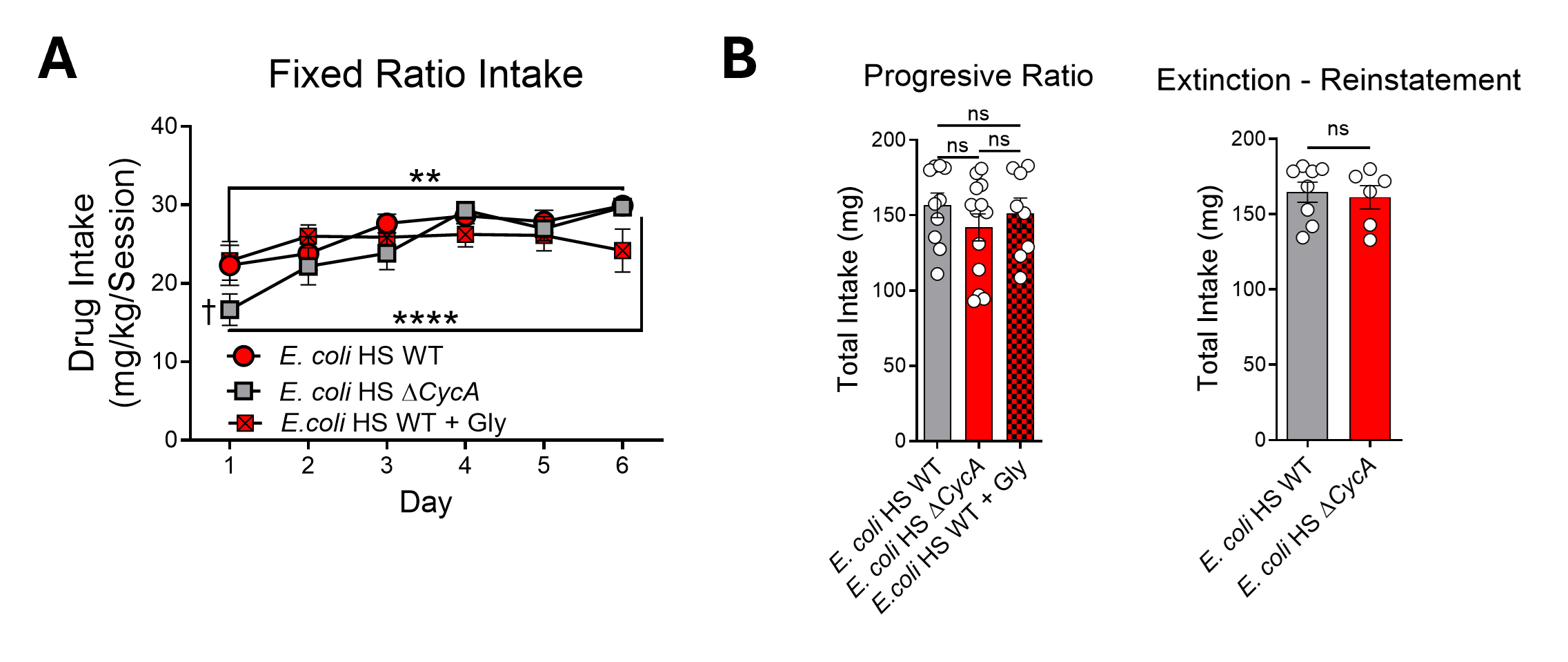

Supplement: Supplementary Material — Supp files.zip [file KGMI_A_2693397_SM4543.zip › Supplementary Figure 1.png]

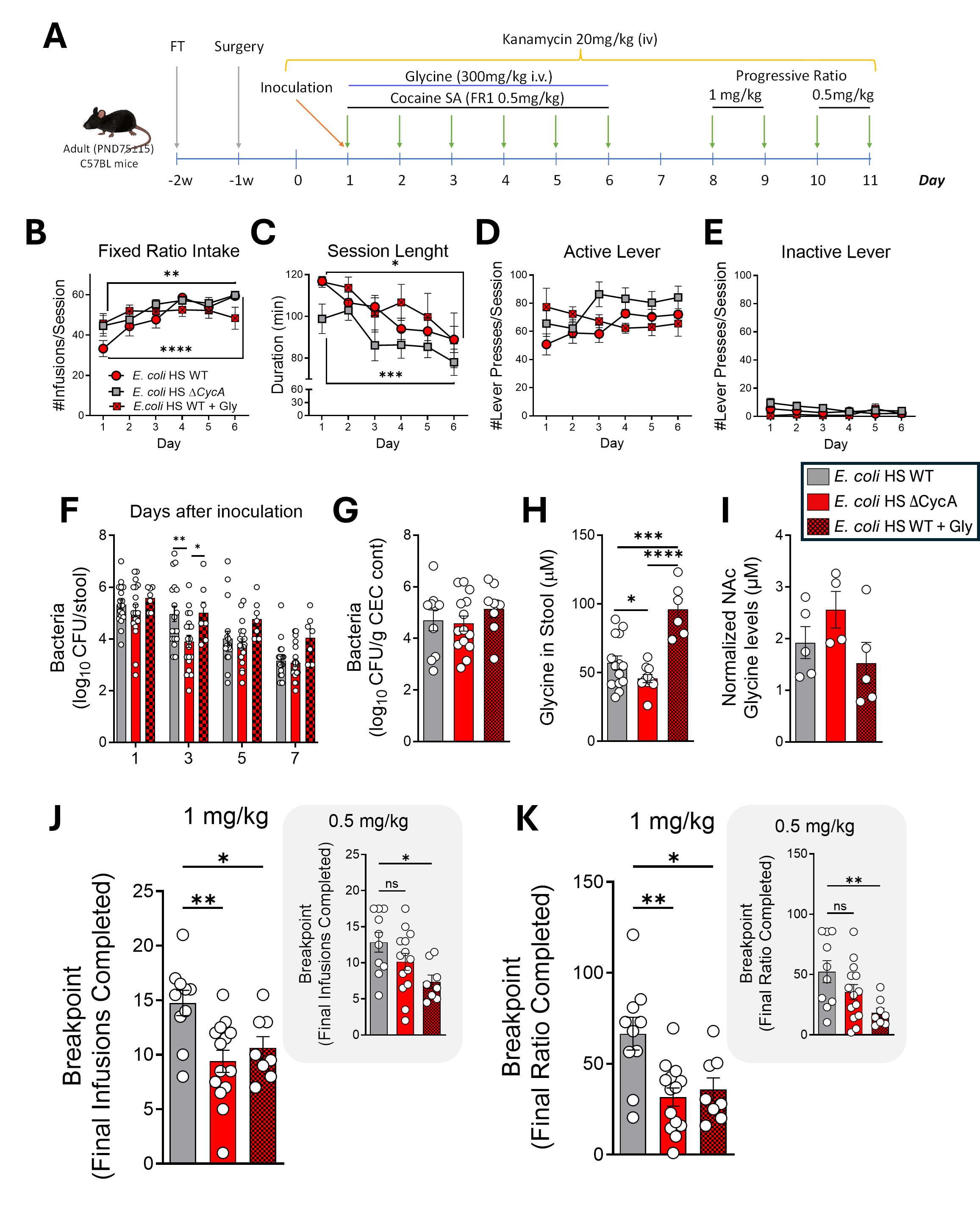

Supplement: Supplementary Material — Supp files.zip [file KGMI_A_2693397_SM4543.zip › Supplementary Figure 2.png]

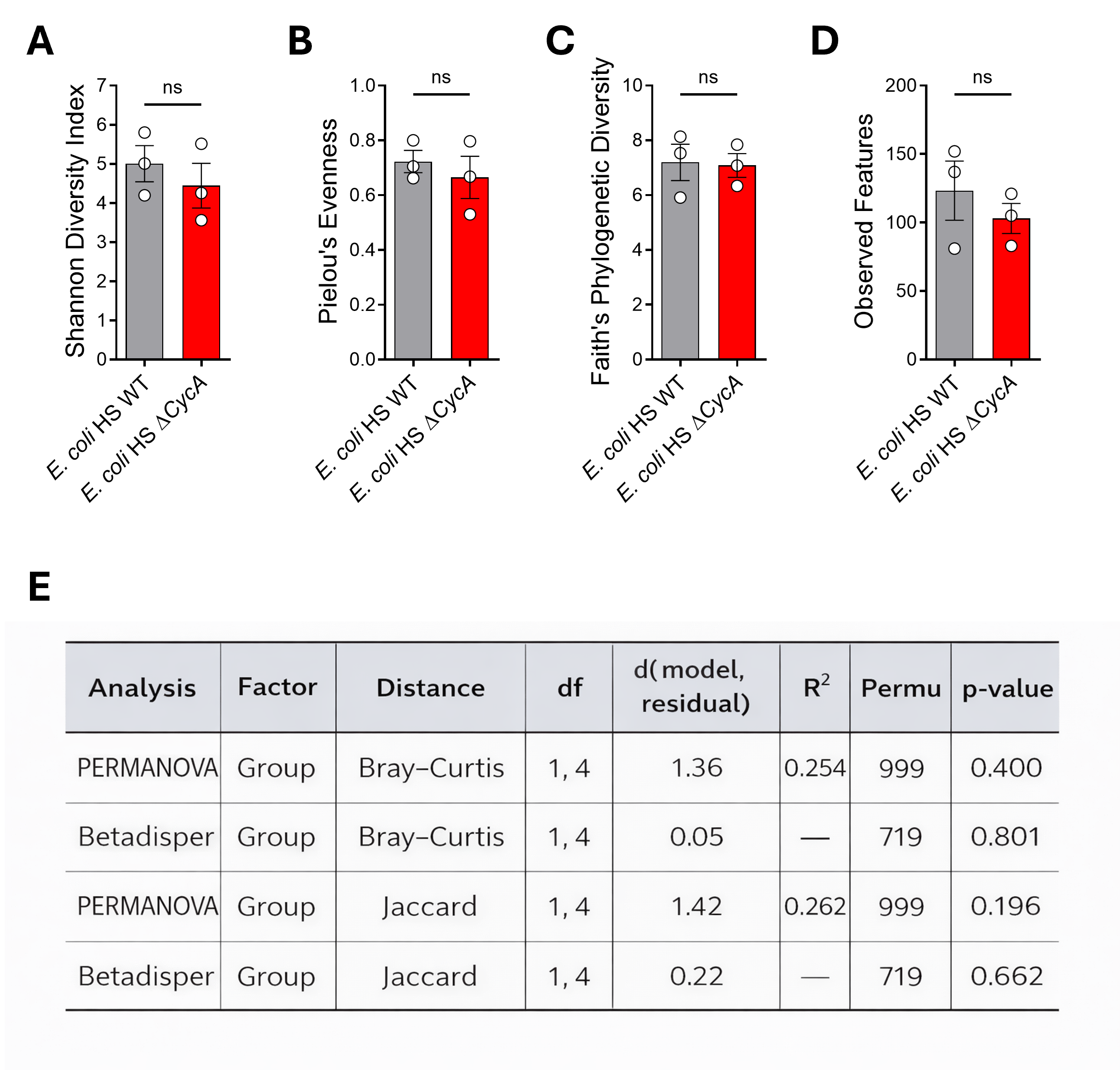

Supplement: Supplementary Material — Supp files.zip [file KGMI_A_2693397_SM4543.zip › Supplementary Figure 3.png]
